# Case report: A rare case of Rosai–Dorfman–Destombes disease after the COVID-19 infection

**DOI:** 10.3389/fmed.2022.1073767

**Published:** 2022-12-19

**Authors:** Pooja Gogia, Fahmina Tanni, Juan Coca-Guzman, Neil Chen, Yiwu Huang

**Affiliations:** ^1^Department of Hematology and Oncology, Maimonides Medical Center, Brooklyn, NY, United States; ^2^Department of Pathology, SUNY Downstate Health Sciences University, Brooklyn, NY, United States

**Keywords:** COVID-19, COVID-19 vaccination, Rosai–Dorfman disease, sinus histiocytosis with massive lymphadenopathy, Moderna vaccine

## Abstract

Coronavirus disease 2019 (COVID-19) caused by severe acute respiratory syndrome coronavirus 2 (SARS-CoV-2) is known to cause immune dysregulation and, therefore, has varied and often rare presentations. Rosai–Dorfman–Destombes disease (RDD) is an unusual non-Langerhans cell (non-LC) histiocytosis presenting with massive lymphadenopathy and various systemic symptoms. A 55-year-old Asian-American woman with no significant medical history or recent use of new drugs initially presented with cervical lymphadenopathy and urticarial rash 1 week after receiving the COVID-19 messenger RNA (mRNA) vaccine (Moderna, mRNA-1273) against SARS-CoV-2. The biopsy of the skin rash was consistent with a drug reaction. Approximately 2 months later, she developed mild flu-like symptoms and was diagnosed with a COVID-19 infection. Her symptoms were mild and self-resolving. Approximately 3 months later, she developed a generalized patchy erythematous rash on the face and the body that gradually worsened; diffuse lymphadenopathy involving the bilateral cervical, axillary, and inguinal areas; and constitutional symptoms. Laboratory results were consistent with lymphopenia, anemia, and an elevated sedimentation rate. Supraclavicular lymph node biopsy showed Rosai–Dorfman disease with a marked polyclonal plasmacytosis. She was started on a tapering dose of corticosteroids and showed clinical improvements over the next few weeks. Herein, we present a rare case of a histiocytic disorder that developed after contracting the SARS-COV2 infection in the event of receiving a recent mRNA COVID vaccination.

## Introduction

Rosai–Dorfman–Destombes disease (RDD) is a rare systemic macrophage-related disorder characterized by sinus histiocytosis and massive lymphadenopathy (1). The etiology of RDD is not fully understood and varies across the spectrum of phenotypes (2).

Severe acute respiratory syndrome coronavirus 2 (SARS-CoV-2) is a virus that has caused an outbreak of the illness Coronavirus disease 2019 (COVID-19), which has been declared a public health emergency by the World Health Organization (WHO), an agency of the United Nations responsible for international public health (3). In individuals infected with COVID-19, there is a dysregulation of the adaptive immune response that leads to a cytokine storm and widespread multi-organ involvement (4). A recent case report described a sinus histiocytosis with massive lymphadenopathy (Rosai–Dorfman disease) that developed after contracting a COVID-19 infection (5).

Messenger RNA (mRNA) vaccines against SARS-CoV-2 have been widely used, and several case reports of patients with vaccination-induced lymphadenitis have been published. In a retrospective study, unilateral lymphadenopathy was identified at the vaccination site in about 44% of patients after they received the COVID-19 vaccination with the aid of imaging studies, and persistent lymphadenopathy was observed at 43 weeks after vaccination (6). We identified a case report of the development of cutaneous RDD after 10 days of receiving Pfizer vaccination against COVID-19 (7). A few cases of histiocytic necrotizing lymphadenitis after vaccination against COVID-19 have been reported in the literature (8). Another case of Langerhans cell (LC) hyperplasia associated with the COVID-19 mRNA vaccine has also been reported (9). Here, we present a rare, biopsy-confirmed case of RDD after receiving the COVID-19 mRNA vaccine and then contracting a COVID-19 infection.

## Case report

**Figure 1:**
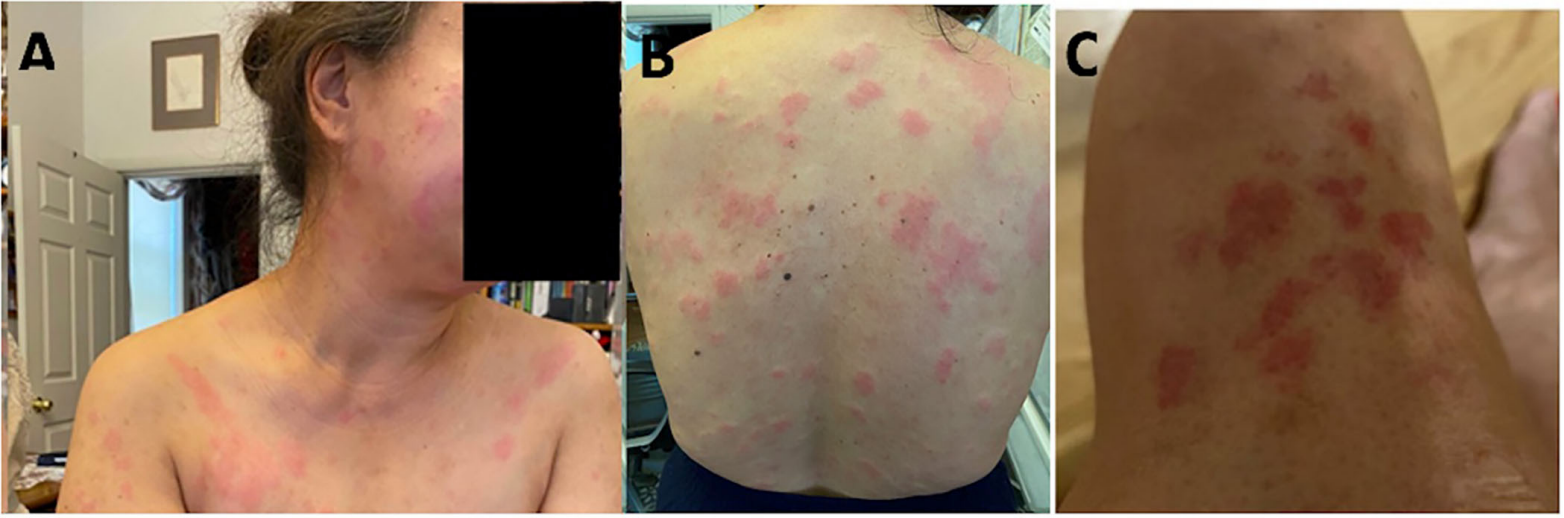
Illustration of a patchy erythematous plaque-like rash on the face **(A)** and an erythematous plaque-like rash on the body **(B,C)**.

**Figure 2:**
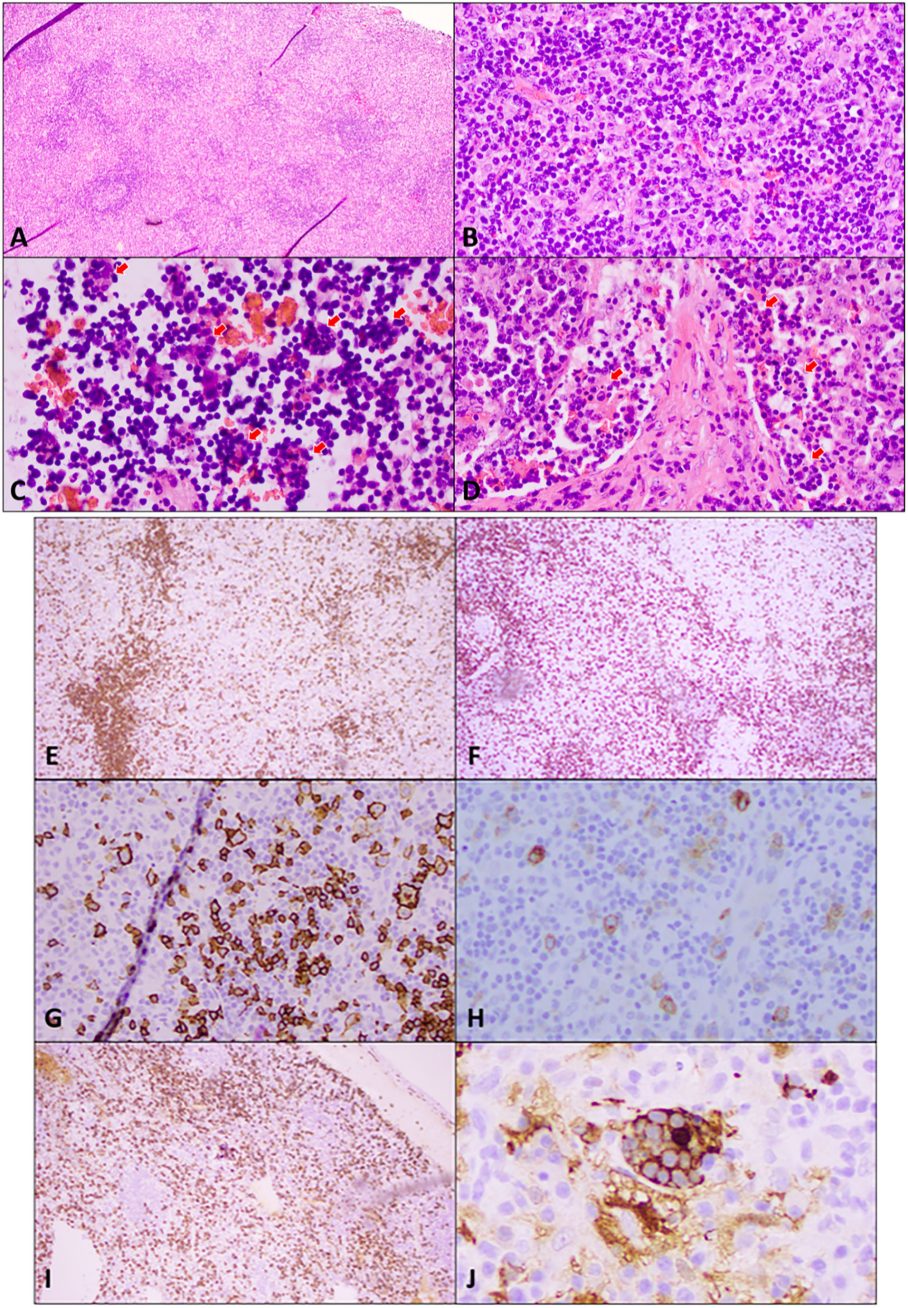
Excisional biopsy of an enlarged supraclavicular lymph node. **(A)** Hematoxylin and eosin (H&E) staining at low power (4×) shows widely spaced reactive follicles with a prominent paracortex expansion. **(B)** H&E medium power (20×) view demonstrates a mixed small and large atypical lymphocytic infiltrate in the paracortex against the background of abundant plasma cells, immunoblasts, and histiocytes. **(C)** H&E high power (40×) view of cells in sinuses shows numerous histocytes with vacuolated nuclei and abundant cytoplasm engulfing lymphocytes and other cells in a process termed emperipolesis (red arrows). **(D)** H&E high power (40×) view of the stain demonstrates emperipolesis (red arrows) in sinuses with adjacent areas of fibrosis. Immunohistochemical studies: **(E)** A low power (10×) view of CD20 immunostaining shows disrupted reactive follicles and scattered large atypical B-lymphocytes. **(F)** A lower power (10×) view of CD3 immunostaining highlights the admixed paracortical T-cells. **(G)** A medium power (20×) view of the CD20 immunostaining is positive in atypical lymphocytes. PAX 5 also stains the large atypical cells (not shown). **(H)** CD30 immunostaining (20× power) highlights the large atypical lymphocytes in interfollicular areas, which were negative for CD15 (not shown). This makes Hodgkin's lymphoma unlikely. **(I)** CD138 immunostaining (10×) demonstrates the presence of abundant plasma cells, which are also positive for CD79a, MUM1 with polyclonal kappa, or lambda by *in situ* hybridization (not shown). Plasma cells are often associated with Rosai–Dorfman disease. **(J)** Areas of emperipolesis are better demonstrated with S100 stain (high power, 60×), which highlights histiocytes with an abundant cytoplasm that have engulfed lymphocytes and other cells (emperipolesis). Engulfed cells do not stain but are outlined by S100 staining when engulfed.

Our patient is a 55-year-old woman of Chinese descent with no known medical history who initially presented to the office with chief complaints of painless swelling in the left side of the neck and a generalized rash persisting for the past 1 month. She is a lifetime non-smoker, has never abused alcohol, and has never used illicit drugs. No recent prescription or over-the-counter (OTC) drug or herbal supplement was used. Her only distant medical history was infectious mononucleosis at 24 years of age. She has a history of allergic reactions to cefaclor and reported developing an itchy rash. The patient reported to be healthy until she received her COVID-19 vaccine booster shot with Moderna's mRNA vaccination (mRNA-1273) against SARS-CoV-2 on the left arm. Prior vaccination history was uneventful. Approximately 4 days after the vaccination, she developed left arm pain and swelling at the site of injection. Approximately 1 week later, she developed swelling on the left side of the neck, and 2 weeks later, she developed a diffuse urticarial rash. She underwent a skin biopsy of a rash about a month later since presentation in the left mid-back that showed a perivascular cell infiltrate with eosinophils consistent with a drug reaction. Blood work performed on the same date showed a white blood cell (WBC) count of 2.5 × 10^9^/L, a hemoglobin level of 10.9 g/L, and a platelet count of 296,000/mm^3^. Absolute lymphocyte count (ALC) was 1.79 × 10^9^/L, absolute neutrophil count (ANC) was 0.54 × 10^9^/L, MCV was 71 femtoliter, red blood cell (RBC) count was 5.04 × 10^9^/L, and red cell distribution width (RDW) was 15.8%. Her comprehensive metabolic panel was within normal limits. Approximately 15 days later, after the biopsy, she developed a fever and mild cough and was tested positive for SARS-COV-19; however, her symptoms resolved completely within a few days. Repeat blood work done a month later since contracting COVID-19 was also consistent with bi-cytopenia with a hemoglobin level of 9.6 g/L and a WBC count of 1.8 × 10^9^/L with ANC of 1.16 and ALC of 0.40 × 10^9^/L. Liver transaminases were mildly elevated to alanine transaminase/aspartate transaminase (ALT/AST) of 49/59 units/L. Iron studies were within normal limits. Approximately 3 months later, after her COVID-19 infection, she presented again with a painless rash on her face, mainly in the nose and around the mouth and the chin, extending to the upper chest area. She reported worsening of her cervical lymphadenopathy and progressively worsening of lymphadenopathy in the neck, the axilla, and the groin and also developed a rash that progressed to involve the extremities and the back. She also described constitutional symptoms including fatigue and a weight loss of 4 kg over the last 3 months. She declined experiencing fever, chills, or significant night sweats. On physical examination, vitals were within normal limits except for tachycardia of 102 beats/min. Head and neck examination showed a patchy erythematous plaque-like rash on the face and around the nose, the perioral area, and the chin, extending to involve the upper chest and the arms. A patchy erythematous plaque-like rash was also observed in the back and the upper extremities, as shown in Figure 1. Lymph node examination showed diffuse adenopathy of the bilateral cervical area (levels I to V) more in the left than the right with a maximum size of 2 × 2 cm, palpable lymph nodes in bilateral axillae with a maximum size of 3 × 2 cm more in the left than in the right, and lymph nodes palpable in the bilateral inguinal area. Lymph nodes were soft in consistency and mildly tender. The rest of the examination was within normal limits. Further tests were performed, which showed a WBC count of 2.3 × 10^9^/L (ANC of 1.66 × 10^9^/L, ALC of 0.340 × 10^9^/L, absolute monocyte count of 0.22 × 10^9^/L, and absolute eosinophil count of 0.7 × 10^9^/L), a hemoglobin level of 9 (MCV 75 femtoliter), and an erythrocyte sedimentation rate (ESR) of 46 mm. The chest x-ray was within normal limits. Cytomegalovirus (CMV) IgM and IgG antibodies were positive, Epstein–Barr virus (EBV) IgM antibody was negative, IgG was positive, the hepatitis panel (both B and C) was negative except Hep A IgG, which was positive, and rapid plasma reagin (RPR) was negative. Complement C3 was decreased to 49, and complement C4 was also decreased to 4 mg/dl, and total complement CH50 was <10 units/ml. ANA was positive, ANA titer was 1 to 320, and an ANA pattern of homogeneous and double-stranded DNA antibodies was negative. QuantiFERON TB was indeterminate. Histoplasma antibodies were negative, and toxoplasmosis IgG antibodies were negative. A computed tomography (CT) scan of the neck 1 month later showed extensive bilateral levels of 1–4 lymphadenopathy, the largest of which was the left base of neck level IV, which was approximately 3 × 2.7 × 2.0 cm. A CT scan of the chest with contrast showed bilateral axillary lymphadenopathy. The largest one on the right axilla was 2.6 × 1.2 cm. The largest one on the left axilla was 2.7 × 1.3 cm. There is no mediastinal or hilar lymphadenopathy. There is no pulmonary nodule or mass. She subsequently underwent a left supraclavicular lymph node biopsy. A supraclavicular lymph node biopsy showed atypical interfollicular B-cell proliferation associated with Rosai–Dorfman disease and a marked polyclonal plasmacytosis, as shown in Figure 2. Flow cytometry showed polyclonal B-cells, and T cells demonstrated no pan-T-cell antigen loss. Polymerase chain reaction (PCR) performed for Ig and T-cell receptor (TCR) gamma gene rearrangement showed a clonal rearrangement for Ig and a polyclonal pattern for TCR gamma.

Treatment was initiated with methylprednisolone at a dose of 24 mg a day, which was slowly tapered down over 3 weeks to 8 mg daily. The patient showed clinical improvements, her skin rash improved, and her lymphadenopathy clinically improved by 80%. She also reported improvements in fatigue and exercise tolerance and then continued on a slow taper of steroids over the next 3 weeks. After 2 months of taking steroids, the patient reported a complete resolution of her symptoms.

## Discussion

Rosai–Dorfman–Destombes disease is a rare histiocytic disorder named after Juan Rosai and Ronald Dorfman who analyzed 34 cases under the name sinus histiocytosis with massive lymphadenopathy in 1969 but was first described in 1965 by the French pathologist Pierre Paul Louis Lucien Destombes (2). It is a non-Langerhans cell histiocytosis (LCH) characterized by the accumulation of activated histiocytes within the affected tissues and is associated with a wide clinical spectrum of presentation. It can occur as an isolated disorder or in association with autoimmune, hereditary, and malignant diseases (2). Most patients present with bilateral, massive, and painless cervical lymphadenopathy with or without systemic symptoms. Other lymph nodes that can be involved are mediastinal, axillary, inguinal, and rarely retroperitoneal lymph nodes (10). In our case report, the patient had bilateral cervical, axillary, and inguinal lymphadenopathy associated with fatigue and weight loss. The skin may be involved in 10% of cases, and lesions can be variable but are usually described as painless, non-pruritic nodules, plaques, or erythematous papules that have a coloration varying from yellow to red to brown (11). Our patient had a patchy erythematous rash on the face, around the nose, and on the periorbital area. Other sites of involvement are extranodal, including the central nervous system, the head and the neck, the ophthalmic, intrathoracic, retroperitoneal, genitourinary, gastrointestinal sites, and the bone. Hematological studies may reveal an elevated ESR, normochromic normocytic anemia, leukocytosis (typically neutrophilia), thrombocytopenia, and eosinophilia, although bone marrow infiltration is rare (2). In the abovementioned case report, the patient had microcytic anemia and leucopenia, which are not seen in classically described RDD. The lab results, however, were consistent with an elevated ESR. Her laboratory findings could be explained by the recent COVID-19 infection (12).

The immunophenotype of RDD histiocytes is characterized by cytoplasmic and nuclear S100, fascin, and CD68 positivity. In contrast to LCH, the cells are CD1a-and CD207 negative (13). In the present case report, the patient's cervical node biopsy histopathology was consistent with polytypic plasmacytosis, which is usually observed with RDD. The histopathology was also consistent with the areas of fibrosis rich in S100-positive cells that show evidence of emperipolesis consistent with RDD. IgG4 diseases are often difficult to distinguish from RDD, especially when the hallmark features such as emperipolesis may not be seen in RDD and when obliterative phlebitis and storiform fibrosis might not be abundant in IgG4 diseases. However, in this case, the pathological features of the specimen were diagnostic, and IgG4-positive cells, although increased, are insufficient, neither in their absolute number nor as a percentage, for a diagnosis of IgG4 diseases. In addition, there is also an increase in IgM-positive cells that are more suggestive of hypergammaglobulinemia (14).

Treatment differs depending on the involvement of nodal areas and symptoms. The disease can be self-limiting but can take many months to years for spontaneous resolution, as reported in 20–50% of patients with nodal/cutaneous disease (15). The response to corticosteroids varies, although they have helped in reducing nodal size and symptoms. Other therapies in patients refractory to steroids include immunosuppressants like sirolimus, chemotherapy with cladribine, methotrexate, vinca alkaloids, immunomodulatory agents like thalidomide, lenalidomide, rituximab, and imatinib, and enrollment in clinical trials (2). Immunomodulatory agents have been explored in the treatment of cutaneous RDD. In a recent case report, a patient who developed cutaneous RDD after receiving an mRNA vaccine was successfully treated for 6 months with thalidomide (7). The National Comprehensive Cancer Network (NCCN) guidelines recommend thalidomide as an option for cutaneous RDD (16). According to a few case reports, lenalidomide has a similar response rate and is more tolerable than thalidomide (17, 18). The optimal duration of steroids or other systemic therapies is not determined, although some clinicians recommend 6–12 months of systemic therapy followed by observation as a reasonable approach (2).

Targeted therapies have also been studied. An activating BRAF V600E mutation is seen in 38–64% of LCH and Erdheim–Chester disease (ECD) cases and more frequently in mixed LCH/ECD (16). In RDD, however, no single dominant mutation has been found, though Kirsten rat sarcoma virus (KRAS) and MAP2K1 mutations have been described in 33% of cases of Rosai–Dorfman disease (3, 19). A published case report of a patient with RDD and a *KRAS* mutation responded well to targeted therapy with cobimetinib (20). A phase II trial evaluating the MEK inhibitor cobimetinib, which included 18 adult patients diagnosed with a histiocytic neoplasm (only two with RDD), demonstrated an overall response rate of 89% with a complete response in 72% of patients. After a follow-up of 11.9 months, the median progression-free survival was not reached. The most common adverse events were a decrease in ejection fraction (27.8%), rash (11.1%), and diarrhea (11.1%) (21). Our patient, as mentioned, had an excellent response to corticosteroids within 3 weeks of treatment initiation.

The Coronavirus disease 2019 infection is a world pandemic, and the spectrum of disease manifestations ranges from asymptomatic disease with mild respiratory tract illness to severe pneumonia, acute respiratory distress syndrome (ARDS), multi-organ failure, and death (22). Various rare manifestations and associations of the disease have been reported in various case reports. So far, only one published case report has depicted an association between RDD and COVID-19. The mechanism of action was hypothesized to be an altered immune response and a cytokine storm caused by interferon dysregulation (5). Another case report describing an LCH presenting as a post-COVID-19 multisystem inflammatory syndrome was published (23). Histiocytic disorders, in particular hemophagocytic lymphohistiocytosis have been postulated as an etiology for triggering cytokine storm in patients with severe COVID-19 and both these diseases demonstrate clinical similarities (24, 25).

Various studies on lymph node autopsy reports have shown the histopathological appearance of decreased total lymphocytes with the absence of germinal centers (26), increased reactive plasmablasts in the interfollicular zone, histiocytic hyperplasia with hemophagocytosis (26, 27), and, in rare cases, necrotizing granulomas (28). Cutaneous manifestations have been described in up to 20% of patients with COVID-19, including erythematous rash, widespread urticaria, varicella-like rash, and purpura (29). Histopathologic examinations of skin biopsies revealed a superficial and deep perivascular infiltrate of lymphocytes or neutrophils, with some eosinophils or plasma cells (29). Endothelial cell injury and thrombotic vasculopathy with fibrinoid or inflammatory thrombi are the most frequent dermal vascular lesions (30).

Widespread vaccination against SARS-CoV-2 has been considered the most promising approach to curbing the pandemic. mRNA vaccines have been widely used and show a high degree of immunogenicity, a high safety profile, and high efficacy in inducing immune responses against SARS-CoV-2 (3). Multiple case reports of patients with vaccination-induced lymphadenitis after COVID-19 vaccination have been published, as described in a systematic review (31). Though most of the studies have shown ipsilateral axillary lymphadenopathy as the site of injection, a few case reports have been published to suggest cervical lymphadenopathy after vaccination (32, 33). Reports of lymphadenopathy are more common in those receiving the Moderna vaccine compared to the placebo and typically occur 2–4 days after vaccination (34).

Histopathologically, in these lymph nodes, there will be an expansion of the paracortex with abundant T cells, B cells, immunoblasts, and plasma cells with increased vascularity, which are similar to those seen with acute viral infections or angioimmunoblastic T-cell lymphoma (35). The detection of clonal B-cell IgH gene rearrangements in the abovementioned patient's lymph node may be related to the patient's exposure to viruses or COVID-19 vaccination.

Enlarged lymph nodes after the COVID-19 vaccine may be mistaken for malignancy, necessitating unnecessary biopsies. However, in patients with associated systemic symptoms, skin manifestations, and progressive diffuse lymphadenopathy, it is imperative to perform a biopsy for a definitive diagnosis and further management. Also, variable cutaneous reactions, including a delayed urticarial rash after receiving mRNA vaccines against COVID-19, have been reported, and most of them are mainly self-limited (36).

In our case report, the patient initially developed cervical lymphadenopathy and urticarial rash 1 week after receiving the mRNA COVID-19 vaccination. A few months after contracting mild respiratory symptoms from COVID-19, the patient developed progressive bilateral cervical, axillary, and inguinal lymphadenopathy associated with fatigue, weight loss, and an erythematous rash on the face, the extremities, and the back.

## Conclusion

In conclusion, the pathophysiology of COVID-19 disease and its association with rare histiocytic disease is incompletely understood but can be hypothesized as immune dysregulation associated with the infection. Recently, a few case reports are evolving suggesting associations between contracting SARS-CoV-2 virus and development of histiocytic disorders with varied presentations. Herein, as described we present a rare case of histiocytic disorder Rosai-Dorfman-Destombes disease manifesting months after receiving mRNA vaccine and further after contracting COVID-19 infection.

## Data availability statement

The raw data supporting the conclusions of this article will be made available by the authors, without undue reservation.

## Ethics statement

Written informed consent was obtained from the individual(s) for the publication of any potentially identifiable images or data included in this article.

## Author contributions

PG and YH: concept and design. PG, FT, and YH: data acquisition. PG, JC-G, NC, and YH: drafted the article. All authors contributed to the article and approved the submitted version.
